# Evaluation of neurofilament light chain in the cerebrospinal fluid and blood as a biomarker for neuronal damage in experimental pneumococcal meningitis

**DOI:** 10.1186/s12974-020-01966-3

**Published:** 2020-10-07

**Authors:** Ngoc Dung Le, Lukas Muri, Denis Grandgirard, Jens Kuhle, David Leppert, Stephen L. Leib

**Affiliations:** 1 Neuroinfection Laboratory, Institute for Infectious Diseases, University of Bern, Friedbühlstrasse 51, 3001 Bern, Switzerland; 2 Graduate School for Cellular and Biomedical Sciences (GCB), University of Bern, Bern, Switzerland; 3 Neurologic Clinic and Policlinic, Departments of Medicine, Biomedicine and Clinical Research, University Hospital and University of Basel, Basel, Switzerland

**Keywords:** Pneumococcal meningitis, Neuroinflammation, Neuronal damage, Neurofilament light chain, Serum biomarker

## Abstract

**Background:**

Pneumococcal meningitis (PM) remains a global public health concern and affects all age groups. If acquired during infancy or childhood, permanent neurofunctional deficits including cognitive impairment, cerebral palsy, and secondary epilepsy are typical sequelae of neuronal injury. Determination of patients at risk for the development of brain injury and subsequent neurofunctional sequelae could help to identify patients for focused management. Neurofilament light chain (NfL) is an axonal cytoskeletal protein released upon neuronal injury into the cerebrospinal fluid (CSF) and blood. As little is known about the course of neurofilament release in the course of PM, we measured CSF and serum NfL levels longitudinally in experimental PM (ePM).

**Methods:**

Eleven-day-old infant Wistar rats were infected intracisternally with *Streptococcus pneumoniae* and treated with ceftriaxone. At 18 and 42 h post-infection (hpi), the blood and CSF were sampled for NfL measurements by a single molecule array technology. Inflammatory cytokines and MMP-9 in CSF were quantified by magnetic bead multiplex assay (Luminex®) and by gel zymography, respectively.

**Results:**

In ePM, CSF and serum NfL levels started to increase at 18 hpi and were 26- and 3.5-fold increased, respectively, compared to mock-infected animals at 42 hpi (*p* < 0.0001). CSF and serum NfL correlated at 18 hpi (*p* < 0.05, *r* = 0.4716) and 42 hpi (*p* < 0.0001, *r* = 0.8179). Both CSF and serum NfL at 42 hpi strongly correlated with CSF levels of IL-1β, TNF-α, and IL-6 and of MMP-9 depending on their individual kinetics.

**Conclusion:**

Current results demonstrate that during the peak inflammatory phase of ePM, NfL levels in CSF and serum are the highest among CNS disease models studied so far. Given the strong correlation of CSF versus serum NfL, and its CNS-specific signal character, longitudinal measurements to monitor the course of PM could be performed based on blood sample tests, i.e., without the need of repetitive spinal taps. We conclude that NfL in the serum should be evaluated as a biomarker in PM.

## Background

*Streptococcus pneumoniae* and *Neisseria meningitidis* are the most prevalent causative pathogens for childhood bacterial meningitis (BM) [1] with fatality rates up to 50% in resource-poor settings [2]. Pneumococcal meningitis (PM) acquired during infancy or childhood is associated with a high risk for neurofunctional deficits which profoundly affect the quality of life. One-third of PM survivors display long-lasting neurological sequelae including hearing loss, cerebral palsy, secondary epilepsy, and cognitive impairment [3, 4].

In reaction to the bacterial infection, the host’s immune response causes an excessive inflammation that leads to injury to the central nervous system (CNS) [4, 5]. The brain structural damage induced in the course of PM results also from vasospasms and reduced blood flow that eventually cause cortical necrosis [6] and apoptotic cell death in the hippocampal subgranular zone of the dentate gyrus [7–9]. Additionally, brain-diffuse ischemic axonal injury and Wallerian degeneration have been demonstrated in brain autopsy of human BM and in experimental PM (ePM) [10]. Along with inflammatory signaling molecules like IL-1β, TNF-α, and IL-6, effector molecules such as matrix metalloproteinases (MMPs) are released in high amounts into the cerebrospinal fluid (CSF) [11], promoting blood-brain barrier (BBB) opening and neutrophil recruitment [12, 13] and may exert as well direct toxicity on neurons [14, 15]. While their levels are linked to the likelihood for long-term sequelae [11, 14], their relevance as biomarkers in clinical practice is limited, as they reflect disease activity only in CSF, but not in the serum. Hence, longitudinal spinal taps would be required to use them for disease monitoring. Functionally, they relate to neuronal damage only indirectly and no biofluid marker has been established in BM that measures neuronal damage itself.

Neurofilaments (NFs) are a major component of the axonal cytoskeleton and consist of three subunits: NF light chain (NfL), medium chain, and heavy chain. Upon axonal damage, NFs are released into the extracellular space and eventually into the CSF and blood. Their increased levels can be used as a biomarker of neuronal damage [16], indicative of actual disease activity and predicting later disease course in neurodegenerative diseases (multiple sclerosis [17], Alzheimer’s and Parkinson’s disease [18], Huntington disease [19], and amyotrophic lateral sclerosis (ALS) [20]), traumatic brain injury [21], and viral and autoimmune encephalitis [22].

With the advent of the single-molecule array (Simoa) technology, the sensitivity for quantitation of NfL has been increased to a degree that levels in serum or plasma can be accurately quantified [23]. This technical progress, the specificity of the signal as of exclusive neuronal origin and the strong correlation between CSF and serum NfL demonstrated in numerous studies, made it possible to use a serum instead of CSF-based analysis. Thus, longitudinal assessment as an inherent prerequisite to specifically monitor CNS disease activity and drug response can be implemented based on blood tests whose acquisition is far less invasive than repetitive lumbar puncture [24].

Only one study has evaluated NFs as a biomarker for neuronal injury in BM across an array of infectious agents. CSF concentrations of NF heavy chain were increased by 85% (22/26) children with BM; peak levels in patients with neurological sequelae were significantly higher than in those without [25].

Present results are the first to investigate the course of NfL in ePM. We hypothesized that serum NfL is a useful biomarker to monitor disease activity in BM, as its kinetics accurately correlated with those in CSF, as well as with typical inflammatory mediators upregulated in ePM.

## Methods

### Infecting organism

A clinical isolate of *Streptococcus pneumoniae* (serotype 3) was cultured overnight in brain heart infusion (BHI) medium, diluted tenfold in fresh, prewarmed BHI medium, and grown for 5 h to reach the logarithmic phase. The bacteria were centrifuged for 10 min at 3100 × *g* at 4 °C, washed twice, and resuspended in saline (NaCl 0.85%). The resuspended bacterial solution was then further diluted in saline to the desired optical density (OD_570nm_). The inoculum concentration was determined by serial dilution and culturing on Columbia sheep blood agar (CSBA) plates.

### Infant rat model of ePM

All animal studies were approved by the Animal Care and Experimentation Committee of the Canton of Bern, Switzerland (license no. BE 01/18). A well-established infant rat model of PM was used [26, 27]. Eleven-day-old male and female Wistar rat pups together with their dams were purchased from Charles Rivers (Sulzfeld, Germany). The dams were provided with tap water and pellet diet ad libitum. Animals were kept in a room at controlled temperature (22 ± 2 °C) and natural light. Intracisternal injections were performed on pups by injection of 10 μl of the inoculum containing 5.25 ± 3.4 × 10^5^ CFU/ml of living *S. pneumoniae* serotype 3. Control animals received an equivalent volume of saline. PM was confirmed by the quantitative analysis of bacterial titers in the cerebrospinal fluid (CSF) at 18 hpi. Five microliters of CSF was gained by puncture of the cisterna magna, followed by serial dilution and cultivation on CSBA plates. To confirm PM in animals which were not punctured at 18 hpi, the blood was cultivated on CSBA plates. The numbers of animals per independent experiments was determined by a maximum litter size of 14 pups. Given the probability of premature abortion of experiments due to reaching humane endpoints or spontaneous death in infected animals, or unsuccessful infection, experiments were designed with 4 mock-infected and 10 infected animals per experiment. Four independent experiments were performed, for a total of 56 animals. Animals without confirmed PM were excluded (*n* = 9). All animals received ceftriaxone (100 mg/kg, i.p., twice daily [b.i.d.]) at 18 and 24 hpi. With the dosing regimen applied in the present study, therapeutic concentrations are maintained until the end of the experiment, so there is no resurgence of bacterial infection at later time points. Animals were weighed and clinically scored according to the following scoring scheme (1 = coma, 2 = does not turn upright, 3 = turns upright in >5 s, 4 = turns upright in <5 s, 5 = normal) at 0, 18, 24, and before sacrificing at 42 hpi. Spontaneous mortality was documented. Blood sampling in all animals was performed at 18 hpi by puncturing the facial vein using a 20-gauge needle. Blood (< 10% of total blood volume) was collected in Microvette® 200 Z (clotting activator/serum) (Sarstedt), kept at room temperature for at least 30 min, and then centrifuged for 10 min at 13,000 × *g* at 4 °C. The supernatant (serum) was collected and kept at − 80 °C for later analysis. Following blood sampling, CSF samples of half of the animals (*n* = 28) were obtained by puncture of the cisterna magna using a 30-gauge needle. CSF samples were only collected from half of the animals to assess if this additional puncture causes an artefactual increase in NfL level. CSF samples were then centrifuged for 10 min at 13,000 × *g* at 4 °C, and supernatants were stored at − 80 °C for later use. Animals were treated with ceftriaxone after the collection of specimens. At 42 hpi, the blood and then CSF were sampled again from all animals, followed by sacrificing with pentobarbital (Esconarkon®, 150 mg/kg, i.p., Streuli Pharma AG, Switzerland). Of note, throughout the experiment, each animal was only punctured once to avoid a possible artificial increase of NfL in CSF and serum by multiple puncturing. Accordingly, if an animal was not punctured successfully or died spontaneously, no CSF could be collected which then resulted in lower datapoints.

### Analysis of cytokine levels in CSF

Cytokines known to be upregulated in PM (IL-1β, TNF-α, IL-6) were assessed using a magnetic multiplex assay (Rat Magnetic Luminex® Assay, Rat Premixed Multi-Analyte Kit, R&D Systems, Bio-Techne) on a Bio-Plex 200 station (Bio-Rad Laboratories) as earlier reported [27]. Five microliters of CSF collected at 18 and 42 hpi were diluted to a final volume of 50 μl. For each sample, a minimum of 50 beads was measured. For samples below the detection limit, a value corresponding to the detection limit provided by the manufacturer was used and multiplied by the dilution factor (IL-1β 2.93 pg/ml; TNF-α 11.5 pg/ml; IL-6 23.2 pg/ml).

### Analysis of NfL levels in CSF and serum

NfL in CSF and serum were analyzed using a SIMOA® immunoassay (Quanterix Corporation, Billerica, MA, USA) as previously described [17]; the antibody pair of this assay is full cross-reactive with murine NfL.

### Analysis of MMP-9 levels in CSF

The amount of MMP-9 in CSF was assessed by gel zymography as reported earlier [11]. Briefly, CSF samples (2 μl) were diluted with 5 μl of 4× sample buffer (0.5 M Tris-HCl pH 6.8, 10% sodium dodecyl sulfate (SDS) 20%, 42.5% glycerol, 0.5% bromophenol blue) and 13 μl of distilled water (dH_2_O) to a loading volume of 20 μl and electrophoresed under non-reducing conditions in 10% SDS-polyacrylamide gels containing type A gelatin from porcine skin (1%; Sigma, Buchs, Switzerland) as proteinase substrate. After electrophoresis for 2 h at 100 V, gels were incubated for 2 × 30 min in SDS-removing buffer (1% Triton X-100), washed 5× with dH_2_O, followed by incubating in incubation buffer (1.5 M NaCl, 0.5 M Tris-HCl pH 7.6, 4% NaN_3_, 10 mM CaCl_2_) for 18 h at 37 °C on a shaker. Gels were then stained with Coomassie blue (0.05% Coomassie blue in 40% methanol, 10% glacial acid, 50% dH_2_O) to visualize the substrate lysis zones of MMP-9 (92 kDa) and MMP-2 (72 kDa). The gelatinolytic activities of MMP-9 and MMP-2 were analyzed using ImageJ software. The amount of expressed MMP-9 was determined as a percentage of that of the constitutively expressed MMP-2 for each sample [26].

### Statistical analysis

Statistical analyses were performed with GraphPad Prism (Prism 7; GraphPad Software Inc., San Diego, USA). Data were first tested for normal distribution with the Kolmogorov-Smirnov test.

To compare the differences between the non-normally distributed data, a non-parametric Mann-Whitney test was used. Correlations were assessed by using the Spearman correlation coefficient. The results are presented as median with interquartile range. A *p* value of 0.05 was considered as statistically significant, with *p < 0.05, **p < 0.01, ***p < 0.001, and ****p < 0.0001.

## Results

### Intracisternal puncture causes a minor increase in CSF NfL levels

In the present ePM model, intracisternal punctures are performed to inoculate *S. pneumoniae* or saline in a first step and to collect CSF samples later. To determine whether this mechanical trauma leads to an artifactual neuronal damage, we measured serum NfL levels of animals before any intervention and compared them with animals at 18 hpi which have been inoculated with saline at baseline.

**Fig. 1:**
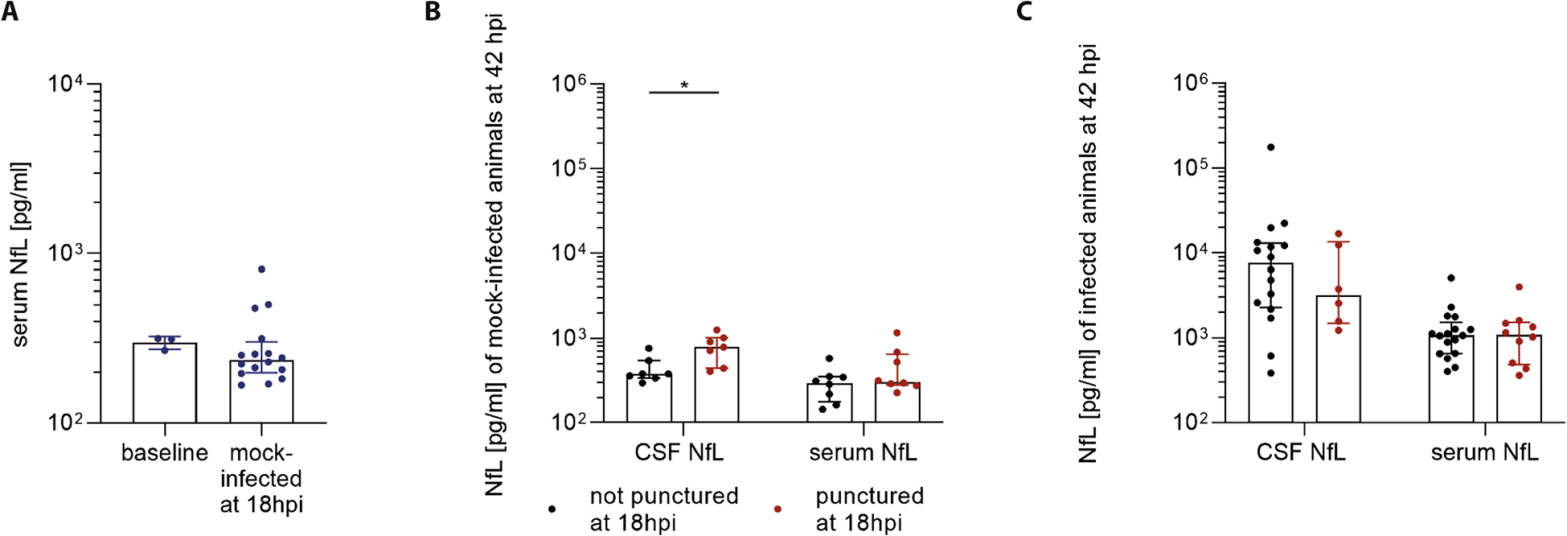
Effect of intracisternal puncture on NfL level. **a** Serum NfL levels of animals (*n* = 3) before any intervention and mock-infected animals at 18 hpi (*n* = 16) were similar. **b** At 42 hpi, mock-infected animals punctured a second time at 18 hpi (*n* = 7) demonstrated higher CSF NfL levels (*p* = 0.01), while there was only a trend for higher serum NfL levels (*n* = 8) compared to non-punctured animals at 18 hpi (CSF *n* = 7; serum *n* = 8). **c** In infected animals, second time punctured (CSF *n* = 6; serum *n* = 10) and not punctured animals (CSF *n* = 16; serum *n* = 17) displayed similar NfL levels both in CSF and serum at 42 hpi

Baseline levels of serum NfL of animals before any intervention (mean ± SD; 299.1 ± 26.4 pg/ml) were not different from those of mock-infected animals at 18 hpi (293.7 ± 168.3 pg/ml) (*p* = 0.18) (Fig. 1a), indicating that the first intracisternal puncture for the inoculation of *S. pneumoniae* or saline had no impact on serum levels. In mock-infected animals undergoing a second intracisternal puncture at 18 hpi for CSF collection, serum levels (471.4 ± 318.6 pg/ml) at 42 hpi increased 1.6-fold compared to those having been punctured only at baseline (299.3 ± 137.2 pg/ml) (*p* = 0.33). Accordingly, CSF levels of NfL showed a 1.8-fold increase in animals having had a second intracisternal puncture (789 ± 303.9 pg/ml) compared to those without (436.7 ± 163.2 pg/ml) (*p* = 0.01) (Fig. 1b). In contrast, at 42 hpi CSF and serum NfL levels in infected animals were more than 2000% (15,319.6 ± 36,745.4 pg/ml) and 300% (1307.5 ± 1062.6 pg/ml) increased, respectively (pooled analysis of 18 hpi punctured and non-punctured) (Fig. 1c), compared to levels in mock-infected animals (Fig. 1b). We conclude that the second intracisternal puncture at 18 hpi did cause a microtrauma, possibly contributing to a < 2% increase of NfL levels in CSF of the total amount observed in the course of ePM, while serum levels in infected animals were not impacted by a second intracisternal puncture. We therefore have pooled the two groups (with and without second intracisternal puncture) for further analyses.

### Animals with ePM have increased CSF and serum NfL concentrations

**Fig. 2:**
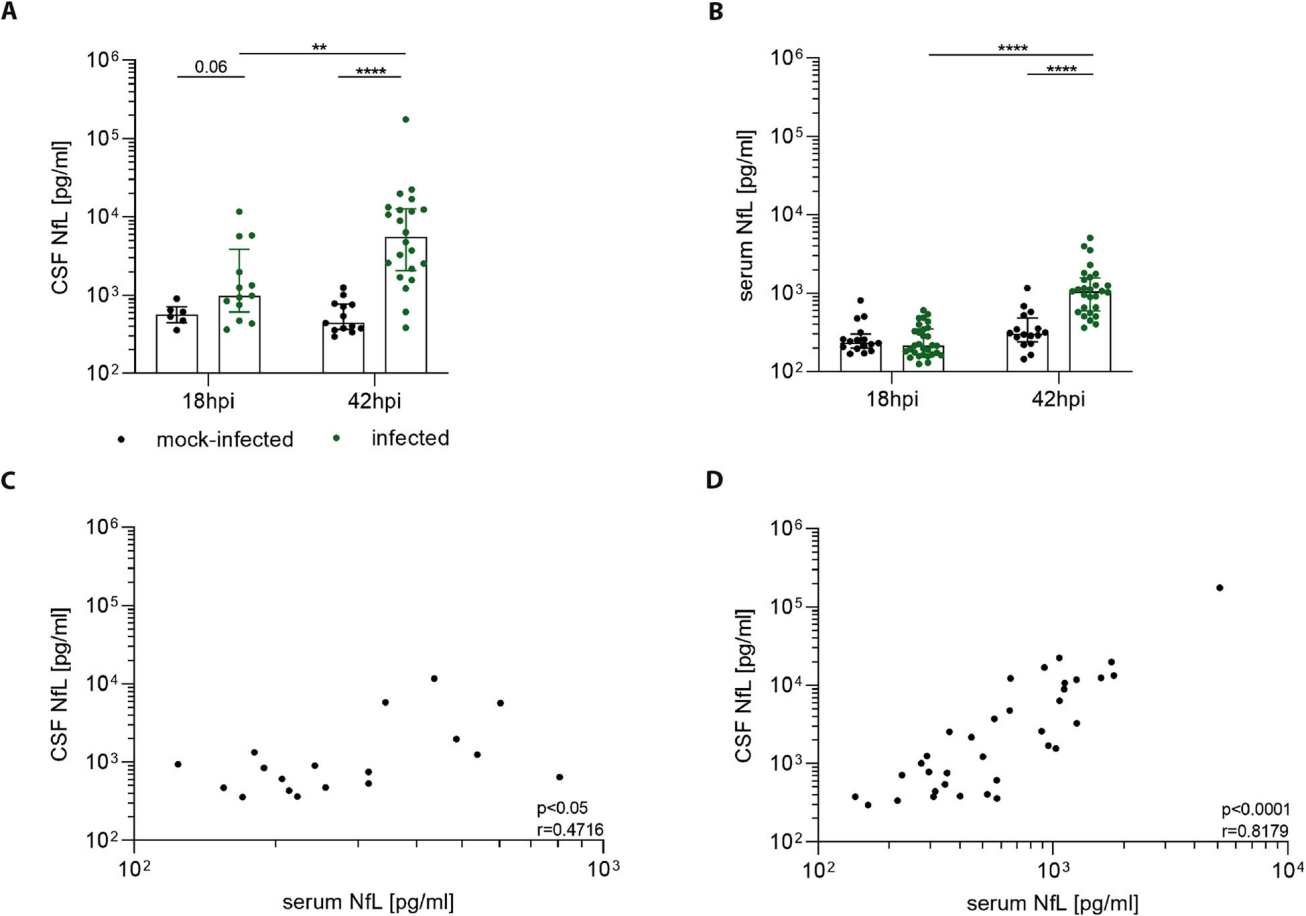
The course of NfL in experimental PM. **a**, **b** At 18 hpi, infected animals displayed non-significantly increased CSF NfL levels (*n* = 13) compared to mock-infected animals (*n* = 6), whereas both displayed similar levels in serum NfL (mock-infected *n* = 16; infected *n* = 30). At 42 hpi, infected animals showed a significant increase of NfL levels in CSF (*n* = 22) and serum (*n* = 28) compared to mock-infected ones (*p* < 0.0001) (CSF *n* = 13; serum *n* = 16). In mock-infected animals, both CSF and serum NfL levels remained stable between 18 and 42 hpi. NfL levels in CSF and serum showed a correlation at 18 hpi (*p* < 0.05, *r* = 0.4716; *n* = 18) (**c**) and at 42 hpi (*p* < 0.0001, *r* = 0.8179; *n* = 35) (**d**)

In the next experiment, we evaluated the time course of NfL increase in infected animals. At 18 hpi, CSF NfL concentrations (2517.1 ± 3336.3 pg/ml) trended to be higher compared to mock-infected animals (590.1 ± 186.9 pg/ml) (*p* = 0.06) and at 42 hpi, they showed a 26-fold higher level (15,319.6 ± 36,745.4 pg/ml) compared to mock-infected controls (590.1 ± 296.4 pg/ml) (*p* < 0.0001) (Fig. 2a). At the same time point, serum NfL levels of infected animals (1362.8 ± 1136 pg/ml) were 3.5-fold higher than mock-infected animals (385.4 ± 253.1 pg/ml) (*p* < 0.0001), while at 18 hpi the levels between the two groups were similar (Fig. 2b). The relative increase of NfL levels over time in infected animals was highly significant in both CSF (*p* < 0.01) and in serum (*p* < 0.0001), whereas those of mock-infected animals remained stable in both fluid compartments (Fig. 2a, b).

At 18 hpi NfL levels in CSF and serum were correlated (*p* < 0.05, *r* = 0.4716) with an average CSF concentration (1908.6 ± 2877 pg/ml) being 6.9-fold higher than that in serum (276.9 ± 144.8 pg/ml) (Fig. 2c). A stronger correlation was also observed at 42 hpi (*p* < 0.0001, *r* = 0.8179) with average CSF levels (9848.6 ± 29768 pg/ml) being 9.8-fold higher than in serum (1007.3 ± 1029 pg/ml) (Fig. 2d).

### Kinetics of cytokines and MMP-9 upregulation in the course of ePM

**Fig. 3:**
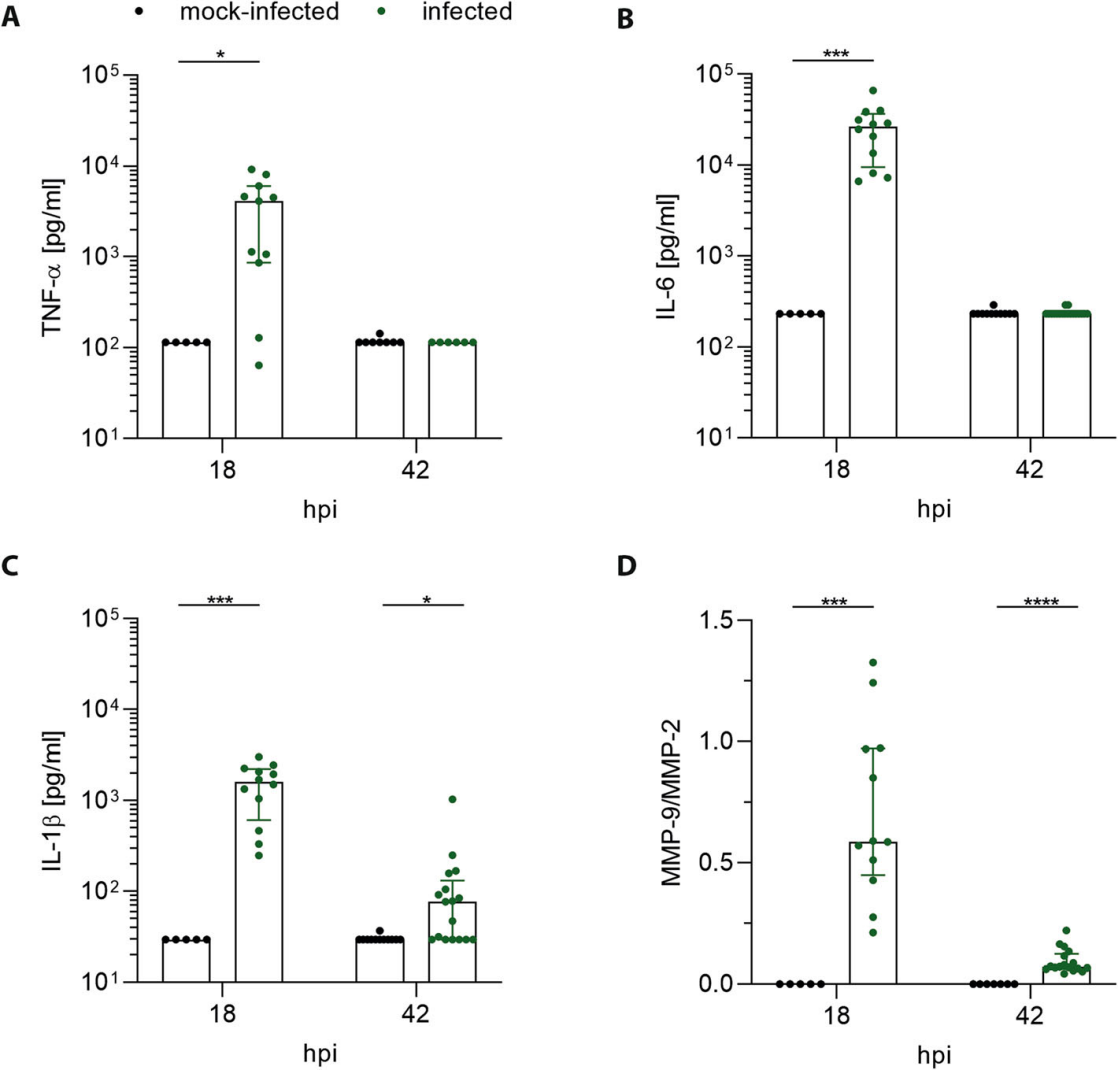
Inflammatory CSF cytokines and MMP-9/MMP-2 levels during ePM. At 18 hpi before treatment with ceftriaxone, infected animals displayed significant higher TNF-α (*p* = 0.01) (*n* = 11) (**a**), IL-6 (*p* < 0.001) (*n* = 12) (**b**), IL-1β (*p* < 0.001) (*n* = 12) (**c**), and MMP-9/MMP-2 (*p* < 0.001) (*n* = 12) (**d**) levels compared to mock-infected animals (*n* = 5 for all cytokines and MMP9-/MMP-2). At 42 hpi, concentrations of TNF-α (**a**) and IL-6 (**b**) were similar in mock-infected (*n* = 8; *n* = 11) and infected animals (*n* = 6; *n* = 18) whereas IL-1β (*p* = 0.02) (*n* = 17) (**c**) and MMP-9/MMP-2 (*p* < 0.0001) (*n* = 17) (**d**) were still higher in infected compared to mock-infected ones (*n* = 12; *n* = 7)

We measured the dynamic change of inflammatory cytokines TNF-α, IL-6, and IL-1β and of MMP-9 during the acute phase of ePM and correlated values with those of NfL. The concentrations of all cytokines were upregulated in infected animals at 18 hpi compared to mock-infected animals (Fig. 3a–c) but decreased at 42 hpi to similar levels as in mock-infected ones for TNF-α and IL-6; only IL-1β remained higher in infected versus mock-infected animals (*p* = 0.02) (Fig. 3c). The relative amount of MMP-9 (MMP-9/MMP-2 index) followed a similar kinetic with increased levels in infected compared to mock-infected animals at 18 hpi (*p* < 0.001) and 42 hpi (*p* < 0.0001) (Fig. 3d).

Cytokine levels correlated among each other at 18 hpi (all *p* < 0.0001; Suppl. Fig. 1). This was not the case for the correlation of cytokine levels with MMP-9, except for IL-1β at 18 hpi (*p* = 0.04, *r* = 0.5126) and a trend at 42 hpi (*p* = 0.07, *r* = 0.3940) (Suppl. Fig. 2).

Table 1 shows the correlation of CSF cytokine and MMP-9 levels with NfL at different time points and for both fluid compartments. At 18 hpi, TNF-α, IL-6, and IL-1β correlated with CSF NfL but not with serum NfL. In addition, cytokine levels at 18 hpi correlated with both CSF and serum NfL at 42 hpi, with the exception of TNF-α that the set significance level for CSF NfL was missed by a margin. At 42 hpi, levels of IL-1β correlated with NfL in both fluid compartments. Moreover, MMP-9 levels at 18 and 42 hpi were correlated with serum NfL at 42 hpi, while for CSF NfL this correlation became significant only at 42 hpi.

**Table 1:**
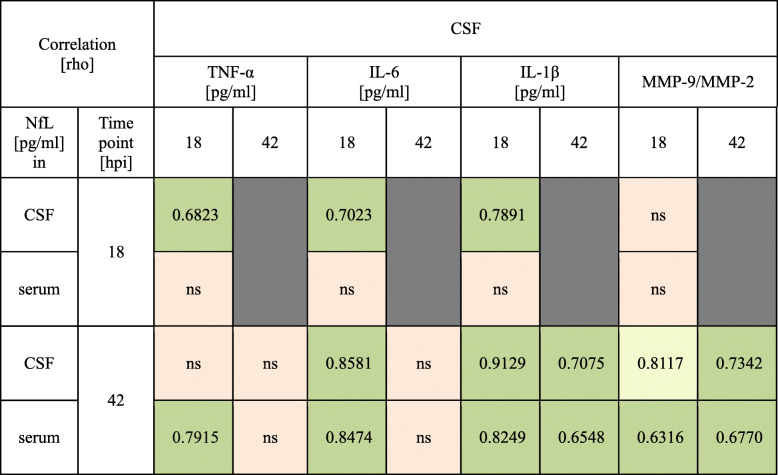
Correlation of inflammatory cytokines, MMP-9, and NfL in CSF and serum. Green = *p* < 0.05, yellow = a trend towards *p* < 0.05, red = non-significant

## Discussion

Neurofilament light chain has been established in recent years as a neuron-specific marker of damage within the CNS, reflecting disease activity and therapy response in many neurological diseases [23, 28] and is now at the doorstep of clinical application [24]. However, little is known about whether NfL could be clinically useful in the same capacity in BM.

In the present study, we investigated the course of NfL release in ePM, a well-characterized model closely replicating the pathology of human PM. Here, neuronal damage is usually more severe and acute than in most other neurological diseases, but there is currently no biomarker established to specifically measure this injury directly. Increased concentrations of neuron-specific enolase (NSE) have been found in patients [29] and in animal models [30], but this marker is not specific for neuronal damage in CSF, as neuroendocrine tissues outside the CNS, most prominently in the lung, are important cellular sources. S100B is an astrocyte-derived marker of brain damage [31]. Several studies have reported an increase of S100B in CSF of human BM [32, 33] and in a rabbit model of PM [31]. However, S100B concentrations already peaked at 20 h after experimental infection and have a short serum half-life which may explain a high percentage of negative scores [34]. These factors may elucidate why both markers have not become standard tests in clinical settings, together with the fact that repetitive lumbar puncture to rely on CSF measurements is impracticable.

In contrast, the strong correlation of serum and CSF measures of NfL makes it an ideal marker to monitor CNS-based pathology. Present results demonstrate that NfL is upregulated in CSF and serum during ePM and confirm the close relationship between the two fluid compartments. Further, we showed that the increase of pro-inflammatory cytokines TNF-α, IL-6, and IL-1β in CSF during an early stage of the disease correlated with the later degree of NfL release. Accordingly, CSF levels of MMP-9, an effector molecule of tissue damage that is released in the function of cytokine expression [35, 36], were also quantitatively linked to the amount of NfL release. The correlation of CSF cytokines and CSF cell counts has been described elsewhere [37, 38] while no correlation has been demonstrated between CSF cell counts and CSF NF in patients with BM [25].

Because our kinetic CSF study required cisterna magna punctures at two different time points in the same animal, we had to evaluate whether this procedure does lead to artifactual neuronal damage interfering with disease-related levels of NfL. The baseline intracisternal puncture with saline did not cause an increase in serum NfL concentration as compared to animals without any intervention. As our primary interest is to demonstrate the value of NfL as a serum-based marker for ePM and to limit the use of animals, we therefore refrained from performing referring CSF measurements.

At 42 hpi, mock-infected animals punctured for a second time at 18 hpi for CSF collection showed a small increase of CSF NfL concentrations compared to animals punctured only at baseline, while serum levels were unaffected. We conclude that this intervention, likely in combination with a local inflammation due to the first puncture, caused a minor neuronal damage. Because the amount of artifactually induced NfL release was less than 2% of that found in ePM at 42 hpi, we conclude that the NfL levels measured are almost exclusively reflective of the infection-related pathology.

NfL can reach the systemic circulation by the bulk flow through the ventricular system and then exiting into the subarachnoid space where it is then reabsorbed into the blood stream [39]. In addition to this, current results suggest that in the initial phase of the disease, the BBB and blood-CSF barrier (BCSFB) are mainly intact, and only small amounts of NfL can reach the circulation. As a consequence of the BBB opening in the course of disease, the correlation of NfL in CSF and serum becomes stronger over time, and the correlation of serum levels with those of CSF cytokines seems to reflect the degree of neuroinflammation in blood circulation.

In ePM, TNF-α, IL-6, and IL-1β appear early, prior to neutrophil recruitment, indicating that they are released by brain-resident cells [40, 41]. TNF-α was shown to appear already 4 h after infection, reaching the peak at 12 h and persisting until 20 h after infection [26]. While the surge of cytokines released at this stage seems to directly lead to later neuronal damage, the induction of MMP-9 by these stimuli may be a further enhancing factor. MMP-9 induces degradation of the BBB and therefore facilitates leukocyte extravasation [12, 40]. Additionally, MMP-9 activates and regulates cytokine signaling in a positive feedback loop, enhancing the excessive inflammation [42, 43] and is suggested to play a role in glial and neuronal cell death [44] and development of neurological sequelae after PM [14].

During BM, microglia are activated by several stimuli including pathogen-associated and damage-associated molecular patterns (PAMPs & DAMPs) and pro-inflammatory mediators, leading to the production of cytokines and chemokines. Nevertheless, excessive microglial activation can result in neuronal damage [45]. In the present study, we did not analyze the microglial status between 18 and 42 hpi. Previously, the microglia status was investigated at the morphological levels at 24 and 42 hpi, showing a transformation of thick ramified to amoeboid, phagocytic phenotype [46]. Since inflammation in the CSF is also resulting from the leucocytic infiltration, it is difficult to determine the specific involvement of microglia. At the transcriptomic level in the cortical tissue, a decrease of the component of the Toll-like receptor pathways between 24 and 72 hpi was observed while there was an increase of the expression of genes guided by the clearance of tissue damage and regeneration [46].

This study has several limitations. We used an experimental model where bacteria are directly injected into the CSF which therefore does not reflect the natural course of the disease. Nonetheless, this method showed a higher success rate compared to methods of hematogenous infection, where successful meningitis is only developed in around 50% [47, 48]. In addition, it is a useful model for studying the interaction between the host and the pathogen as well as the complications resulting from the disease. Next, we only focused on the disease phase of PM up to 42 hpi, with inflammatory cytokine concentrations showing their peak between 18 and 24 hpi in infected animals and a resolution to almost background levels at 42 hpi [27, 49]. The kinetic of NfL after 42 hpi and during the recovery processes has not been determined yet. Further longitudinal investigations with later time points will be needed to assess the kinetic of NfL release and its correlation with chronic clinical sequelae of PM.

In the present study, the degree of cortical damage was limited and gross cortical necrosis was not observed (data not shown) and NfL release resulted mainly from brain-diffuse damage. The correlation of NfL release with defined morphological features of a neuronal damage is the next step underway in our laboratory.

Nevertheless, this study indicates that NfL is a very sensitive marker able to quantitate neuronal damage before it manifests with the most severe stage of typical PM pathology.

## Conclusion

During the acute phase of BM, the upregulation of inflammatory mediators causes neuronal damage. Although inflammatory mediators could be used as predictors, they are not a direct indicator of neuronal injury. Furthermore, a clinically useful biomarker for BM needs to be blood-based to avoid the invasive procedure of lumbar puncture. Serum NfL is a sensitive biomarker which reflects neuronal damage with high specificity. Given the strong correlation between CSF and serum, current results provide the conceptual evidence for the use of NfL as a promising biomarker for the evaluation of neuroprotective agents in BM.

## Supplementary information

**Additional file 1: Figure S1**. At 18 hpi, inflammatory cytokines – TNF-α, IL-6 and IL-1β – correlated with each other (n=17).

**Additional file 2: Figure S2**. IL-1β significantly correlated with MMP-9 at 18 hpi (p=0.04, r=0.5126; n=17) (**A**), but showed only a trend at 42 hpi (p=0.07, r=0.3940; n=22) (**B**).

## Data Availability

The datasets used and/or analyzed during the current study are available from the corresponding author on reasonable request.

## Abbreviations

BBB: Blood-brain barrier
BCSFB: Blood-CSF barrier
BHI: Brain heart infusion
BM: Bacterial meningitis
CFU: Colony-forming unit
CNS: Central nervous system
CSBA: Columbia sheep blood agar
CSF: Cerebrospinal fluid
DAMPs: Damage-associated molecular patterns
ePM: Experimental pneumococcal meningitis
hpi: Hours post-infection
MMP: Matrix metalloproteinase
NF: Neurofilament
NfL: Neurofilament light chain
NSE: Neuron-specific enolase
PAMPs: Pathogen-associated molecular patterns
PM: Pneumococcal meningitis
Simoa: Single molecule array
