# Supplementary figures and images for: Evaluation of neurofilament light chain in the cerebrospinal fluid and blood as a biomarker for neuronal damage in experimental pneumococcal meningitis
Source: J Neuroinflammation. 2020 Oct 7;17:293. doi: 10.1186/s12974-020-01966-3 (PMC7539528; doi:10.1186/s12974-020-01966-3)

**A**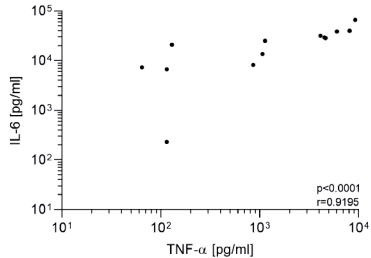**B**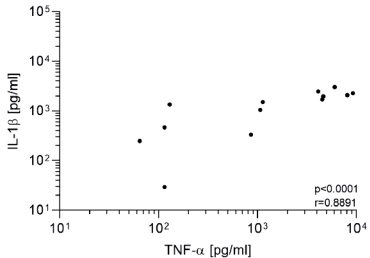**C**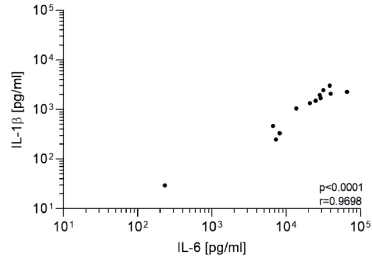

Supplement: Supplementary file 1 — Additional file 1: Figure S1. At 18 hpi, inflammatory cytokines – TNF-α, IL-6 and IL-1β – correlated with each other (n=17). [file 12974_2020_1966_MOESM1_ESM.pdf]

**A**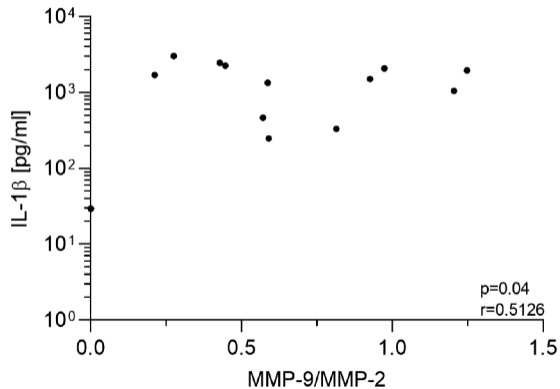**B**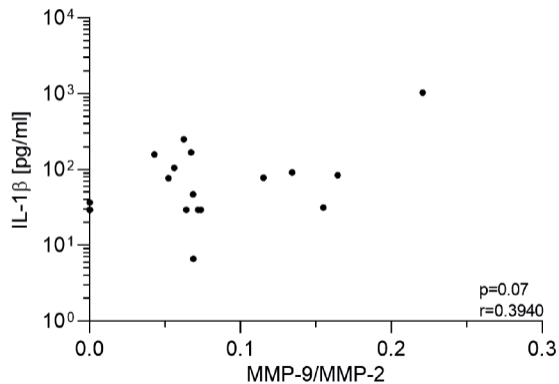

Supplement: Supplementary file 2 — Additional file 2: Figure S2. IL-1β significantly correlated with MMP-9 at 18 hpi (p=0.04, r=0.5126; n=17) (A), but showed only a trend at 42 hpi (p=0.07, r=0.3940; n=22) (B). [file 12974_2020_1966_MOESM2_ESM.pdf]
